# Exploring the Toxicological Impacts of DOTP Exposure on Pulmonary Arterial Hypertension via Network Toxicology, Virtual Knockout, Molecular Docking and Experimental Validation

**DOI:** 10.3390/ijms27188064

**Published:** 2026-09-10

**Authors:** Xiaoying Wang, Xiaoyu Jiang, Zhijun Li, Yingjie Wang, Xiangyang Qin

**Affiliations:** 1 Department of Chemistry, College of Pharmacy, Fourth Military Medical University, Xi’an 710032, China; 202001100@hrbmu.edu.cn; 2 College of Pharmacy, Harbin Medical University, Daqing Campus, Daqing 163319, China; 3 College of Basic Medical Sciences, Harbin Medical University, Daqing Campus, Daqing 163319, China; 4 School of Chemistry and Chemical Engineering, Shaanxi University of Science and Technology, Xi’an 710021, China; 5 State Key Laboratory of Oral & Maxillofacial Reconstruction and Regeneration, School of Stomatology, Fourth Military Medical University, Xi’an 710032, China; 6 National Clinical Research Center for Oral Diseases, Fourth Military Medical University, Xi’an 710032, China; 7 Department of General Dentistry and Emergency, Fourth Military Medical University, Xi’an 710032, China

**Keywords:** pulmonary arterial hypertension, pulmonary artery endothelial cells, DOTP, network toxicology

## Abstract

Environmental factors are significant contributors to the pathogenesis of pulmonary arterial hypertension (PAH). Di-(2-ethylhexyl) terephthalate (DOTP) is a plasticizer widely used in daily life and poses potential health hazards. Nevertheless, the molecular mechanisms underlying PAH development following long-term DOTP exposure remain poorly understood. The aim of this study was to investigate the molecular mechanisms linking DOTP exposure to PAH by integrating network toxicology, virtual knockout, molecular docking and experimental validation methods. We screened four core targets (AKT1, EGFR, KDR, SRC) of DOTP and performed scTenifoldKnk virtual knockout analysis. Functional enrichment indicated that AKT1, EGFR and KDR significantly perturbed pathways related to the regulation of pulmonary artery endothelial cell (PAEC) damage and migration. We subsequently used CCK8, LDH release, RT–qPCR and transwell assays to confirm that DOTP exposure can promote PAEC damage and migration, accompanied by the upregulation of AKT1, EGFR and KDR expression. Finally, molecular docking and molecular dynamics were performed to elucidate the interactions and conformational changes involving DOTP and target proteins. This study reveals the molecular mechanism underlying the occurrence and development of DOTP-induced PAH, providing a new theoretical framework for the investigation of the effects of environmental pollutants on PAEC dysfunction and the pathogenesis of PAH.

## 1. Introduction

Pulmonary arterial hypertension (PAH) is a complex and progressive pulmonary vasculopathy characterized by elevated pulmonary vascular resistance and pulmonary vascular remodeling, ultimately leading to right ventricular hypertrophy and death [1,2]. Its pathophysiology is multifactorial, and includes genetic and congenital factors, underlying cardiovascular and pulmonary diseases, drugs, toxins, and environmental exposures [3]. These factors lead to dysfunction of various types of cells, including pulmonary artery smooth muscle cells (PASMCs), pulmonary artery endothelial cells (PAECs), fibroblasts and macrophages. Among these cells, PAECs are direct targets of pathogenic factors, resulting in cell damage, oxidative stress, inflammatory infiltration and excessive proliferation [4,5,6,7]. Driven by advances in medical research, diverse effective therapeutic approaches have been developed, significantly improving patient prognosis. Nevertheless, the mortality rate of PAH remains relatively high. Therefore, a thorough investigation into the etiological factors of PAH is crucial for improving the quality of life of affected patients. Future investigations should aim to characterize the specific risk factors and molecular mechanisms driving PAH development, especially at the level of endothelial cells, to provide a basis for novel therapeutic interventions.

In recent decades, di-(2-ethylhexyl) terephthalate (DOTP), a major plasticizer, has become widely present in various aspects of daily life, including in car interiors, home decorations, food packaging, and even medical devices [8,9]. Because these products come into direct contact with the human body, such large-scale application may pose potential health risks. For instance, DOTP exposure can promote breast cancer progression by influencing cell proliferation, gene transcription regulation, the tumor microenvironment, and the PI3K-Akt-mTOR pathway [10]. DOTP exerts toxic effects by targeting EGFR, BCL2, NFKB1, CASP3, ERBB2, and mTOR, which further induces chronic spontaneous urticaria [11]. DOTP induces neurotoxicity by promoting oxidative stress, inflammation and apoptotic activation [12]. Allergic respiratory diseases can be triggered by DOTP through immune-related and multi-target mechanisms [13]. However, research investigating the association between DOTP and PAH is lacking. Notably, its structural isomer di-(2-ethylhexyl) phthalate (DEHP) has been confirmed to possess potential carcinogenicity and promote PAH pathogenesis by disrupting key biological processes governing vascular homeostasis. Given their structural similarity, we hypothesized that DOTP might share similar toxic effects and could be a potential risk factor for PAH. Therefore, it is critical to evaluate the relationship between DOTP and PAH and explore the underlying toxicological mechanisms.

Network toxicology represents an emerging field based on biological molecular networks. By integrating multi-omics data and computational models, interactive networks linking compounds, targets, pathways, and toxicity outcomes can be constructed, thereby revealing the mechanisms through which toxicants contribute to disease development [14,15]. The integrated strategy combining network-based target prediction, molecular docking, and experimental validation has been successfully applied to elucidate the mechanisms of various bioactive substances and environmental toxicants [16]. Furthermore, the use of virtual knockout, molecular docking, and molecular dynamics simulation strategies lays a foundation for fully deciphering the toxicological characteristics of this disease and advancing our understanding of its progression. Network toxicology has been applied to various disease studies. Li et al. reported that network toxicology indicated that 6PPDQ exerts hepatotoxic effects mainly through apoptosis, inflammation, and lipid metabolism dysfunction [17]. Qu et al. revealed novel mechanisms underlying 2,2′,4,4′-tetrabromodiphenyl ether (PBDE-47) neurotoxicity using network toxicology and transcriptomics [18]. However, few studies have applied network toxicology to the study of PAH. To date, only two relevant reports have been published [19,20], highlighting the urgent need for further in-depth investigations into the roles of toxicants in the pathogenesis of PAH.

This study employed network toxicology, virtual knockout, and molecular docking techniques, in combination with in vitro experimental validation, to comprehensively investigate the association and potential mechanisms linking DOTP to PAH. This study provides a new theoretical paradigm for understanding the mechanisms underlying PAEC injury caused by environmental pollutants and lays the foundation for developing preventive strategies and targeted therapies for PAH associated with chemical exposure.

## 2. Results

### 2.1. Identification of Potential Target Proteins of DOTP

**Figure 1:**
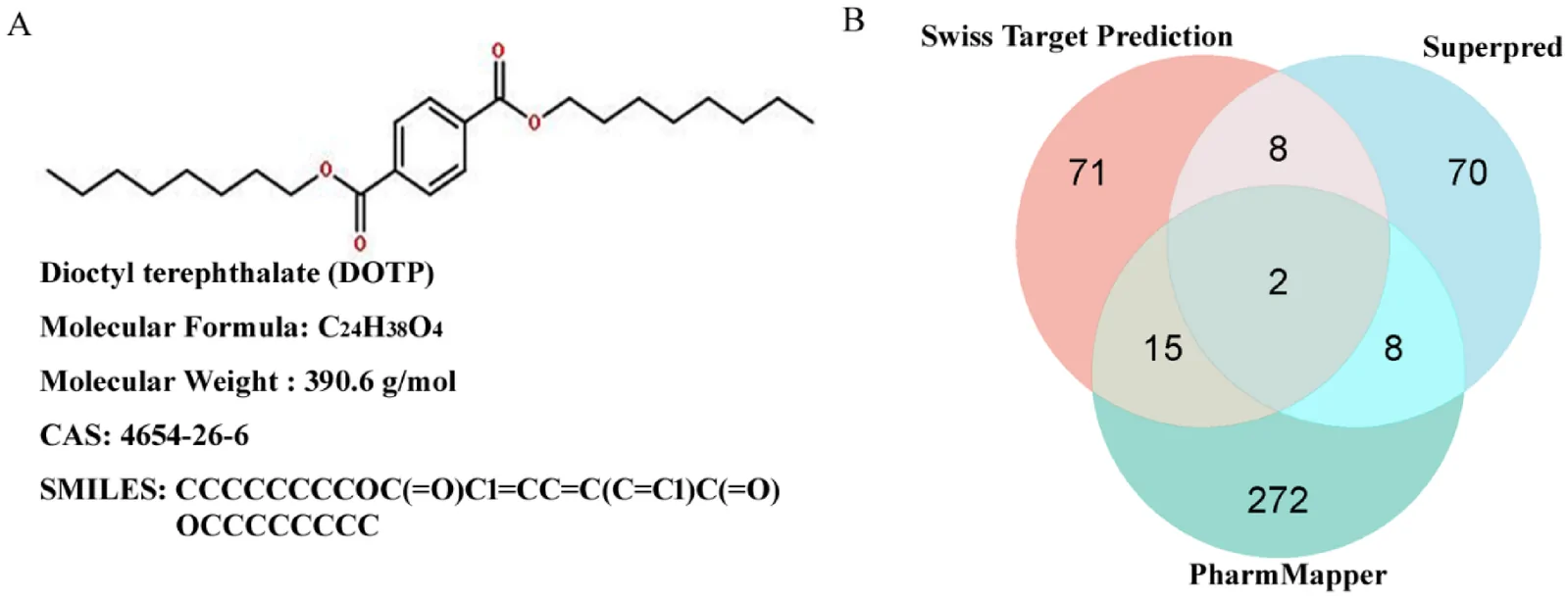
Collection of potential target proteins of DOTP. (**A**) Schematic diagram of DOTP chemical molecules. (**B**) The number of DOTP-related target proteins.

The molecular structure information of DOTP was acquired from the PubChem database (Figure 1A). To comprehensively map its potential biological interactions, Swiss Target Prediction, Superpred, and PharmMapper were used to predict relevant targets of DOTP. A total of 446 potential targets were ultimately obtained after deduplication (Figure 1B).

### 2.2. Identification of PAH-Related Differentially Expressed Genes and Coexpression Network

**Figure 2:**
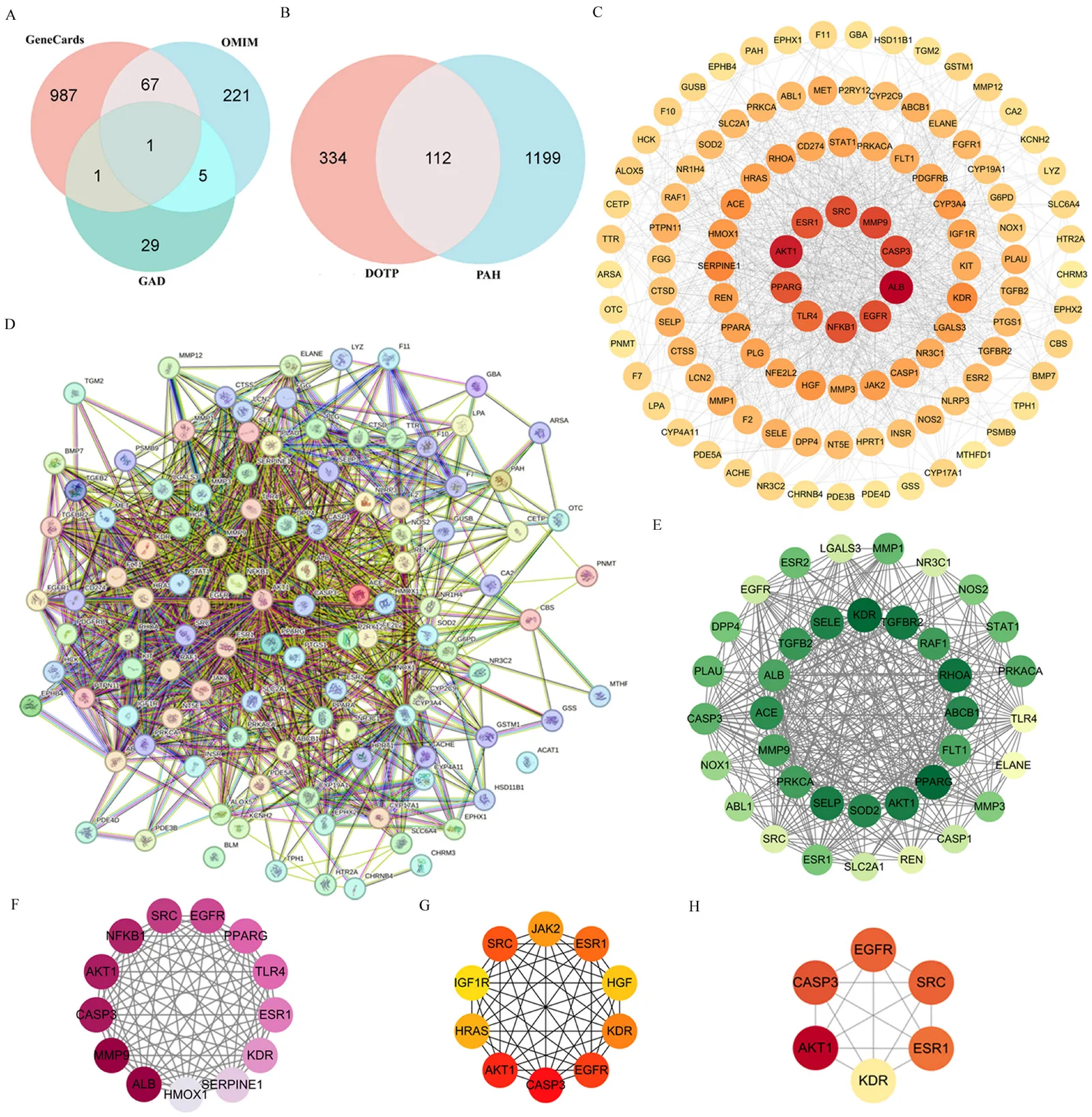
Network toxicology identifying key DOTP–PAH interaction targets. (**A**) The number of PAH-related target proteins. (**B**) Venn diagram of DOTP and PAH targets. (**C**,**D**) PPI network of intersecting targets. (**E**–**G**) Calculation of PPI subnetworks with higher connectivity using MCODE, CytoNCA, and CytoHubba MCC algorithm, with depth of color corresponding to weighted scores. (**H**) Six core targets were identified.

Afterward, PAH-associated targets were retrieved from the GeneCards, GAD and OMIM databases, yielding a total of targets (Figure 2A). A Venn diagram was constructed to illustrate the 113 overlapping targets related to DOTP and PAH (Figure 2B). A protein–protein interaction (PPI) network was constructed using the Cytoscape_v3.7.1 and STRING databases (Figure 2C,D). Further modular analysis using the MCODE, CytoNCA, and CytoHubba MCC algorithms revealed dense interaction clusters among these hub targets, and we identified six core targets: AKT serine/threonine kinase 1 (AKT1), apoptosis-related cysteine peptidase 3 (CASP3), epidermal growth factor receptor (EGFR), estrogen receptor 1 (ESR1), kinase insert domain receptor (KDR), and nonreceptor tyrosine kinase (SRC) (Figure 2E–H).

### 2.3. Target Functional Analysis and Pathway Enrichment Analysis

**Figure 3:**
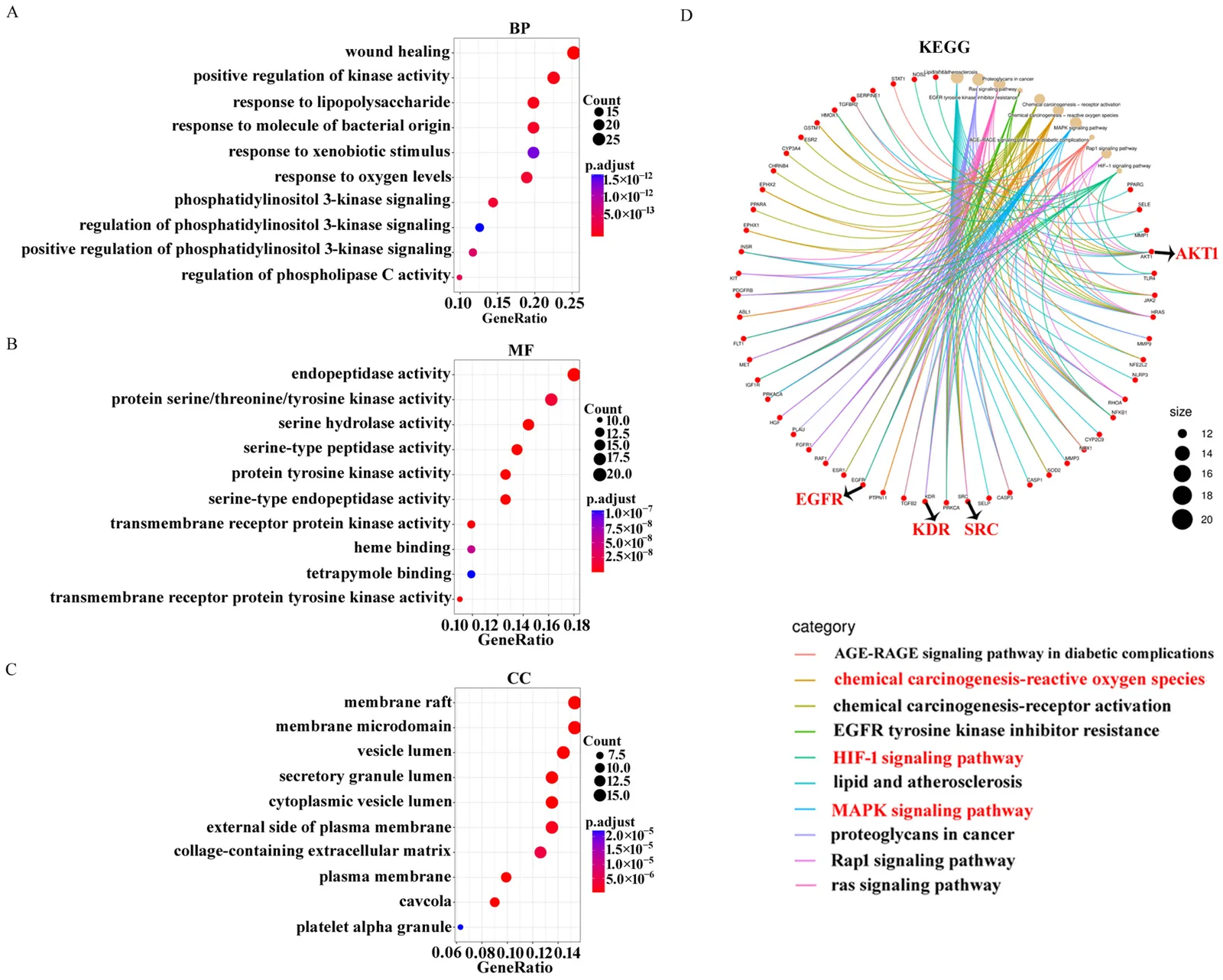
Enrichment analysis of shared targets associated with DOTP exposure and PAH. (**A**–**C**) GO enrichment analysis of the overlapping targets. (**D**) KEGG enrichment analysis of the overlapping targets.

A functional enrichment analysis was conducted on 112 targets, and the top 10 terms from BP, MF and CC were selected and are presented in the form of a bubble chart (Figure 3A–C). The results of the KEGG pathway enrichment analysis are shown in Figure 3D. Three pathways that were significantly related to the pathological process of PAH, namely, the chemical carcinogenesis-reactive oxygen species pathway the HIF-1 signaling pathway, and the MAPK signaling pathway. Moreover, AKT, EGFR, SRC and KDR were identified as proteins common to the three pathways and the six core targets. The above results indicate that DOTP most likely exerts its effects on PAH through these four core targets.

### 2.4. Single-Cell RNA Sequencing Profiling of PAH

**Figure 4:**
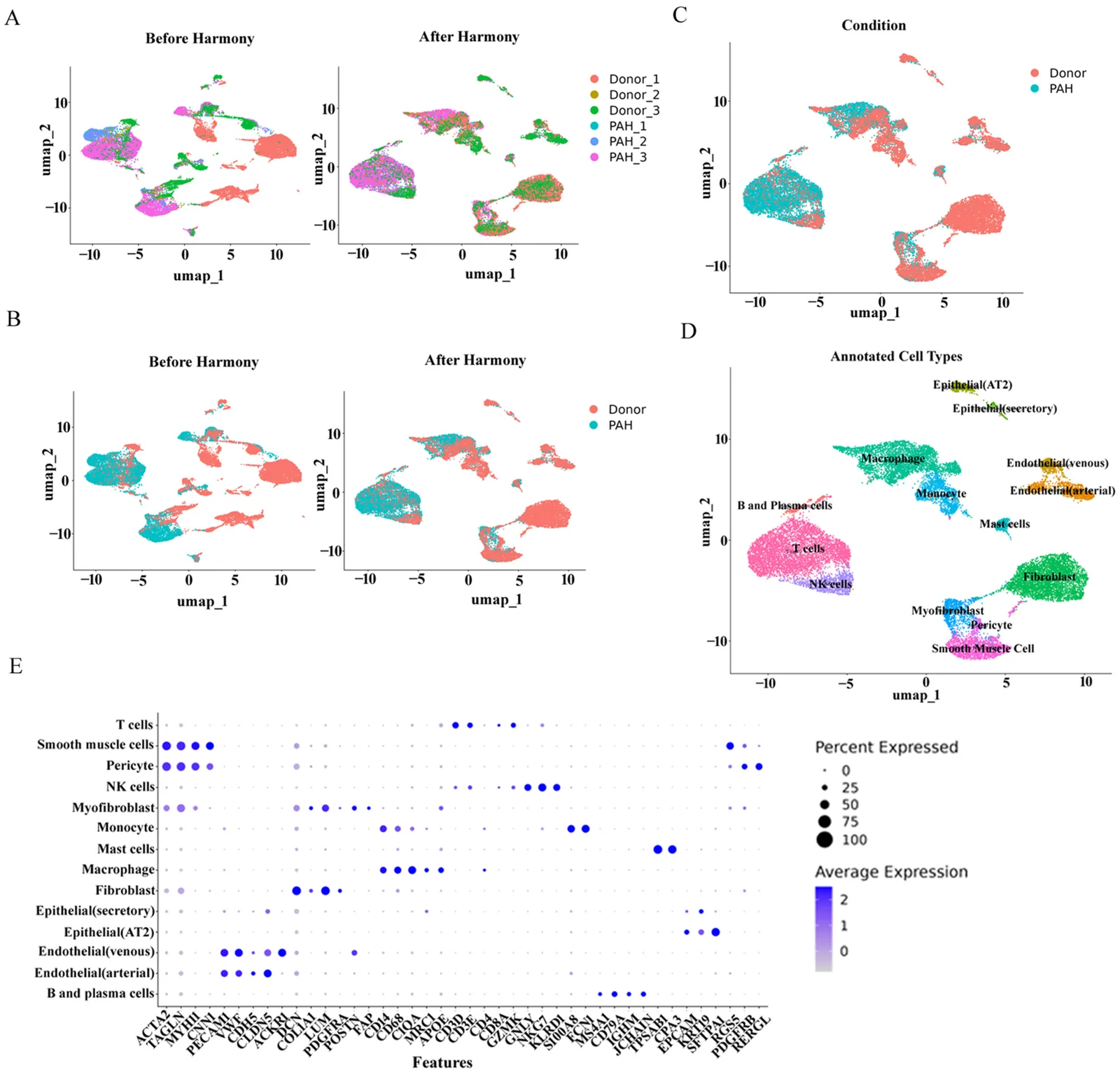
Single-cell RNA sequencing profiling of PAH. (**A**,**B**) UMAP plots showing cells before and after Harmony batch correction. (**C**) Integrated UMAP plot of all cells, colored by condition. (**D**) Annotated UMAP plot of 14 cell types identified in the dataset. (**E**) Dot plot of key marker genes used for cell type annotation, showing average expression and percent expressed per cell type.

To verify the roles of the four core target genes in PAH, we performed an analysis using scTenifoldKnk virtual knockout technology. First, the GSE210248 single-cell sequencing dataset was analyzed in detail, and 22,704 high-quality cells and 19,582 genes were retained after quality control. Batch effects between different samples were successfully eliminated through Harmony batch correction (Figure 4A,B). UMAP dimensionality reduction revealed distinct transcriptomic clustering between healthy donor and PAH patient cells, with overlapping central clusters and group-specific populations enriched on the left (PAH) and right (Donor) sides of the embedding space (Figure 4C). Single-cell clusters were annotated and identified as 14 distinct cell types, including B and plasma cells, T cells, NK cells, macrophages, monocytes, myofibroblasts, pericytes, smooth muscle cells, endothelial cells (venous), endothelial cells (arterial), epithelial cells (AT2), epithelial cells (secretory), fibroblasts, and mast cells (Figure 4D). The expression of the marker genes across these cell types is shown in Figure 4E.

### 2.5. scTenifoldKnk Virtual Knockout Implicates Core Targets in the Regulation of PAEC Migration

**Figure 5:**
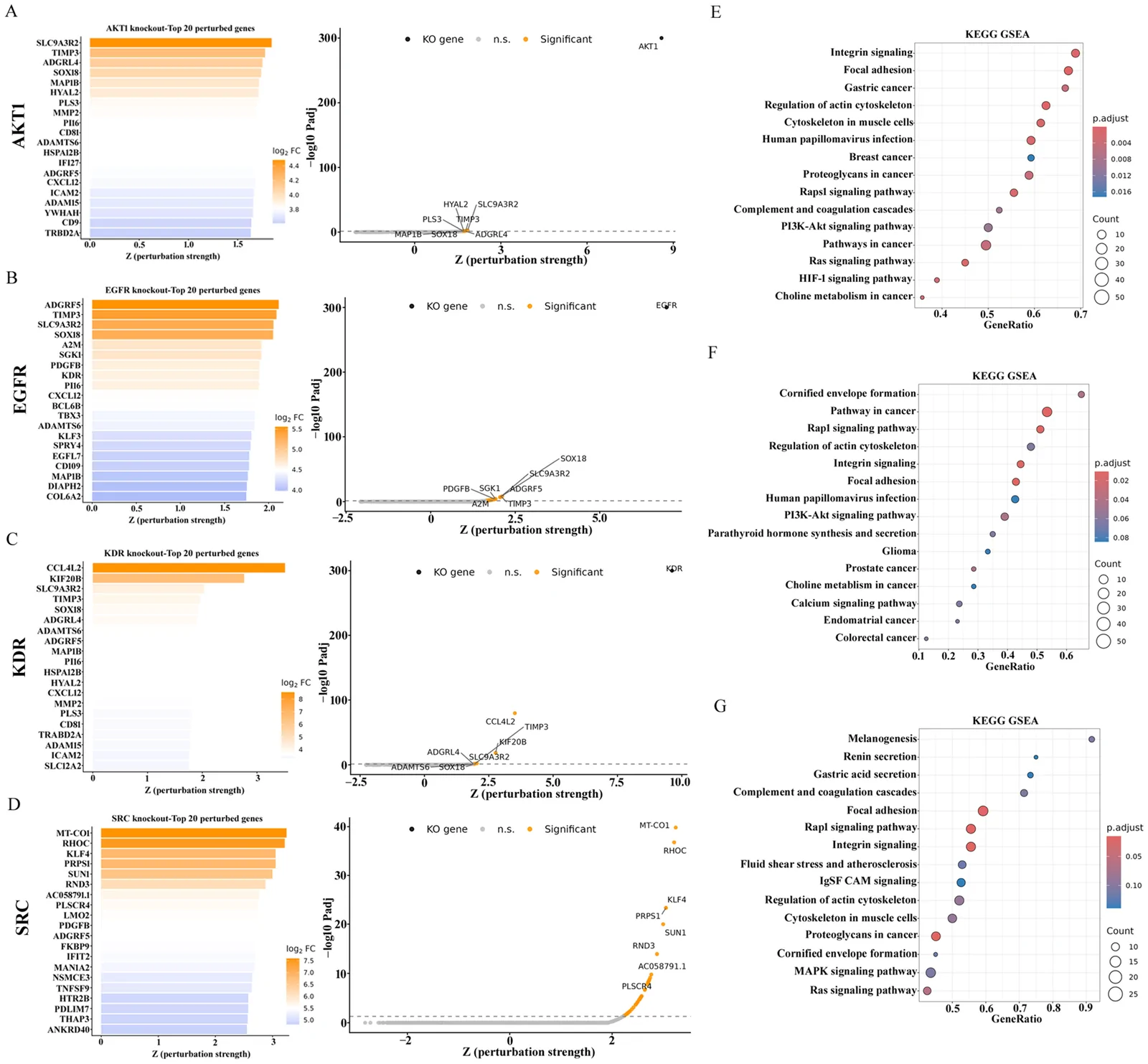
Virtual knockout perturbations and functional enrichment of hub genes. (**A**–**D**) Heatmaps and volcano plots of the top perturbed genes. (**E**–**G**) KEGG enrichment analysis of perturbed genes for each knockout. X-axis: Z-score (perturbation strength). Y-axis: −log_10_ (adjusted *p*-value). Horizontal dashed line: significance cutoff.

To infer the potential regulatory impact of the three core targets on the downstream network, we performed virtual gene knockout perturbations using the scTenifoldKnk package. Successful perturbed gene sets were obtained for AKT1, EGFR, KDR, and SRC. Heatmaps and volcano plots of the top perturbed genes are shown in Figure 5A–D. Specifically, SOX18, ADGRL4, ADGRF5, TIMP3, PDGFB, PI16 and SLC9A3R2 emerged as the most recurrently disrupted genes across AKT1, EGFR, and KDR knockouts, suggesting a core set of downstream targets. By contrast, the analysis for SRC did not yield significant results. GO and KEGG enrichment analyses of the perturbed genes revealed a striking and convergent pattern: successful AKT1, EGFR, and KDR knockouts were significantly and strongly enriched for terms related to the regulation of PAEC focal adhesion and migration (Figure 5E–G).

### 2.6. DOTP Exposure Promotes PAEC Damage and Migration, as Well as Up-Regulated AKT1, EGFR and KDR Protein Levels

**Figure 6:**
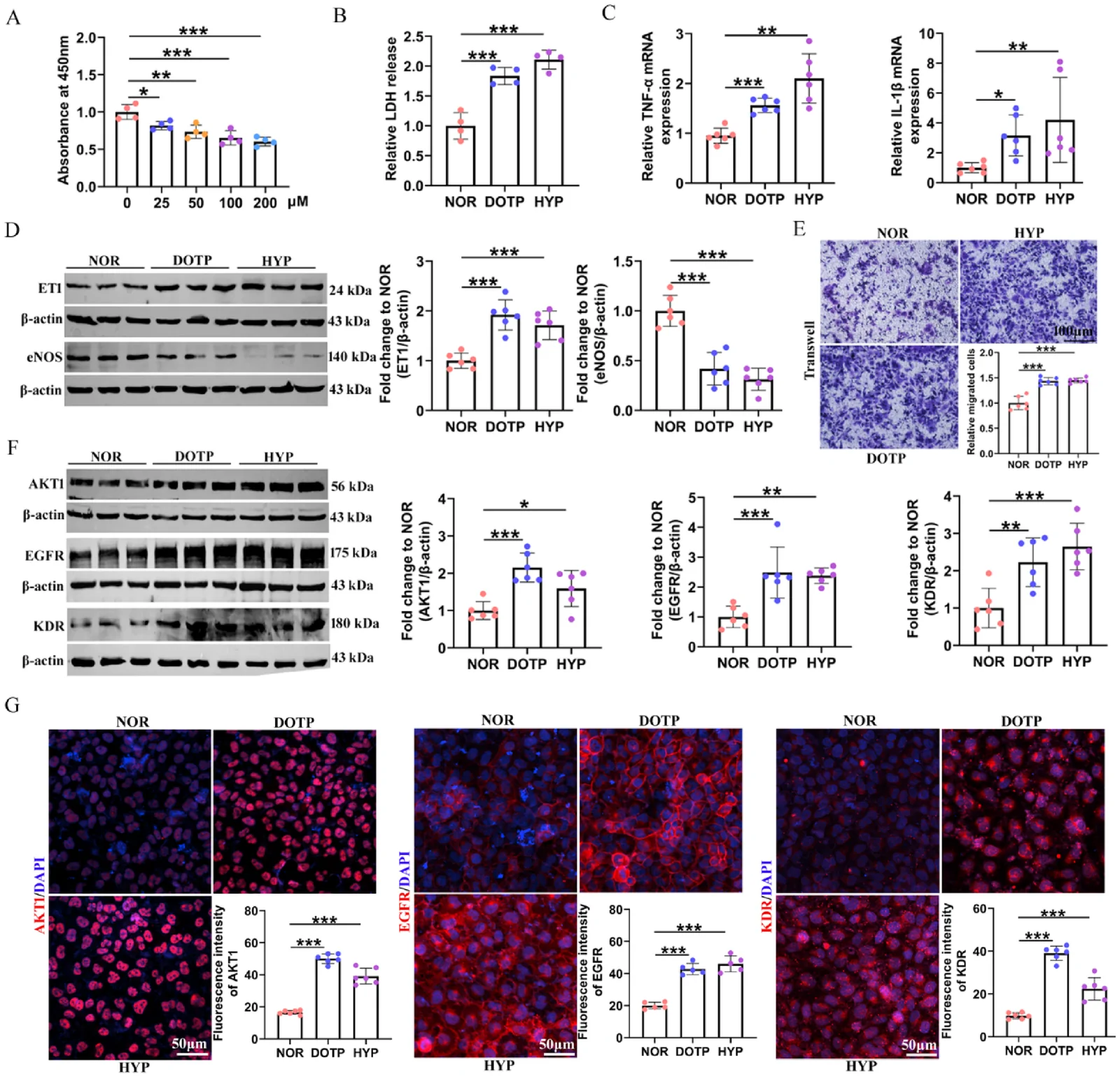
DOTP exposure induces PAEC damage and migration. (**A**) CCK8 assay to screen the optimal DOTP concentration (*n* = 4). (**B**) PAECs toxicity was determined by LDH release assay (*n* = 4). (**C**) RT-qPCR was used to detect the expression of TNF-α and IL-1β in PAECs treatment with DOTP (*n* = 6). (**D**) Western blot analysis was used to verify the expression of ET1 and eNOS (*n* = 6). (**E**) Transwell assay to detect PAEC transwell (*n* = 6). (**F**) Western blot analysis was used to verify the expression of core targets (*n* = 6). (**G**) Core targets expression was further examined by immunofluorescence staining (*n* = 6). Scale bars, 50 μm. All values are presented as the mean ± SD. Statistical analysis was performed with one-way ANOVA. NOR, normoxia; HYP, hypoxic. * *p* < 0.05, ** *p* < 0.01, *** *p* < 0.001.

To verify the regulatory role of DOTP in PAECs, we performed a series of in vitro validation experiments. First, PAECs were cultured with 0, 25, 50, 100, or 200 μM DOTP. The results revealed that the cell viability decreased as the concentration of DOTP increased (Figure 6A). Therefore, 200 μM DOTP was selected for the subsequent experiments. As shown in Figure 6B, the LDH release assay results indicated that compared with hypoxia, DOTP promoted the release of LDH. Moreover, after the PAECs were stimulated with 200 μM DOTP, the levels of TNF-α and IL-1β increased, indicating that DOTP can promote cellular inflammatory damage (Figure 6C). An imbalance between ET-1 and eNOS expression is indicative of endothelial-specific damage. We conducted Western blot experiments to measure the expression of ET1 and eNOS. The results demonstrated that DOTP upregulated ET-1 expression and downregulated eNOS expression, suggesting that DOTP induces endothelial cell-specific injury (Figure 6D). We further investigated the role of DOTP in PAEC migration and reported that DOTP enhanced the migration of PAECs (Figure 6E). To confirm the effect of DOTP on the expression of core proteins, Western blot experiments were performed, which revealed elevated expression of AKT1, EGFR, and KDR in DOTP-treated PAECs (Figure 6F). Similar results are shown in Figure 6G. These results clearly confirmed that DOTP exposure promotes PAEC damage and migration, as well as up-regulated AKT1, EGFR and KDR protein levels. However, all results in this study are derived from in vitro experiments with PAECs. Due to the absence of animal model validation, the conclusions drawn from the current cell-based study cannot be fully extrapolated to complex in vivo conditions. Therefore, in the future, it will be necessary to conduct further in vivo animal studies to verify the toxicological effects of DOTP.

### 2.7. Molecular Docking of DOTP to the Core Targets

**Figure 7:**
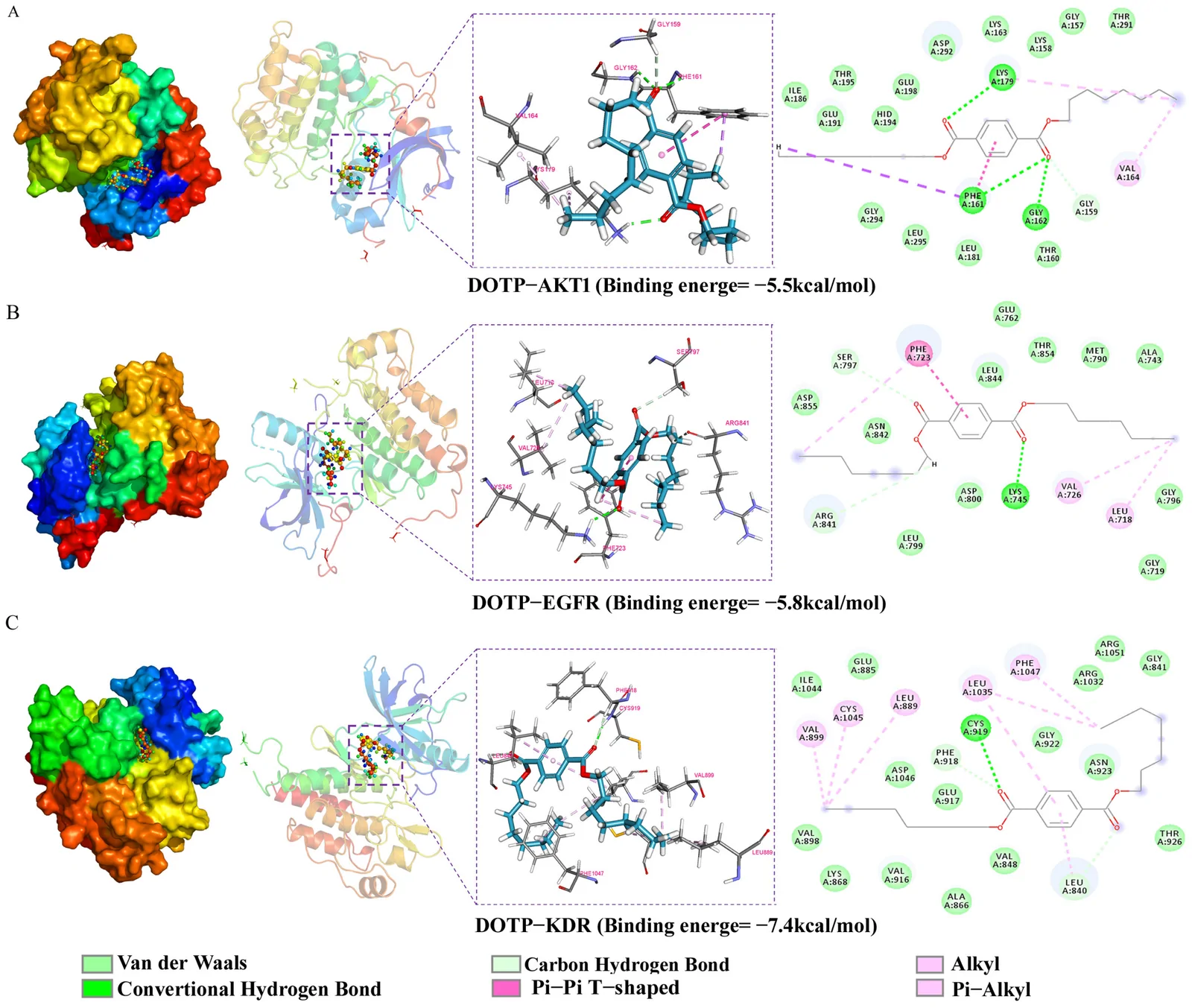
Docking results of core targets and DOTP molecules. (**A**–**C**) Surface representation, cartoon representation, 3D binding mode and 2D interaction diagram for DOTP bound to AKT1 (**A**), EGFR (**B**), and KDR (**C**). Different colors in protein surface structures denote distinct protein structural domains. In 2D interaction plots, light-green represents Van der Waals interactions; dark-green represents conventional hydrogen bonds; pale-green represents carbon-hydrogen bonds; magenta represents Pi-Pi T-shaped interactions; light magenta represents alkyl and Pi-alkyl interactions.

We subsequently conducted molecular docking to explore possible in silico interactions between DOTP and AKT1, EGFR, and KDR. The results showed that DOTP can stably bind to AKT1, EGFR, and KDR, with binding energies of −5.5 kcal/mol, −5.8 kcal/mol, and −7 kcal/mol, respectively, all of which are less than 5 kcal/mol. Structural analysis revealed that DOTP binds to crucial residues of AKT1 through at least one hydrogen bond, together with Pi-sigma, Pi-Pi T-shaped, alkyl and van der Waals forces, which collectively increase the binding strength (Figure 7A). The interactions with EGFR include hydrogen bonding, Pi-Pi T-shaped, alkyl, Pi-Alkyl and van der Waals forces (Figure 7B). The interactions with KDR include hydrogen bond, alkyl, Pi-Alkyl and van der Waals forces (Figure 7C).

### 2.8. Molecular Dynamics Simulations of DOTP to the Core Targets

**Figure 8:**
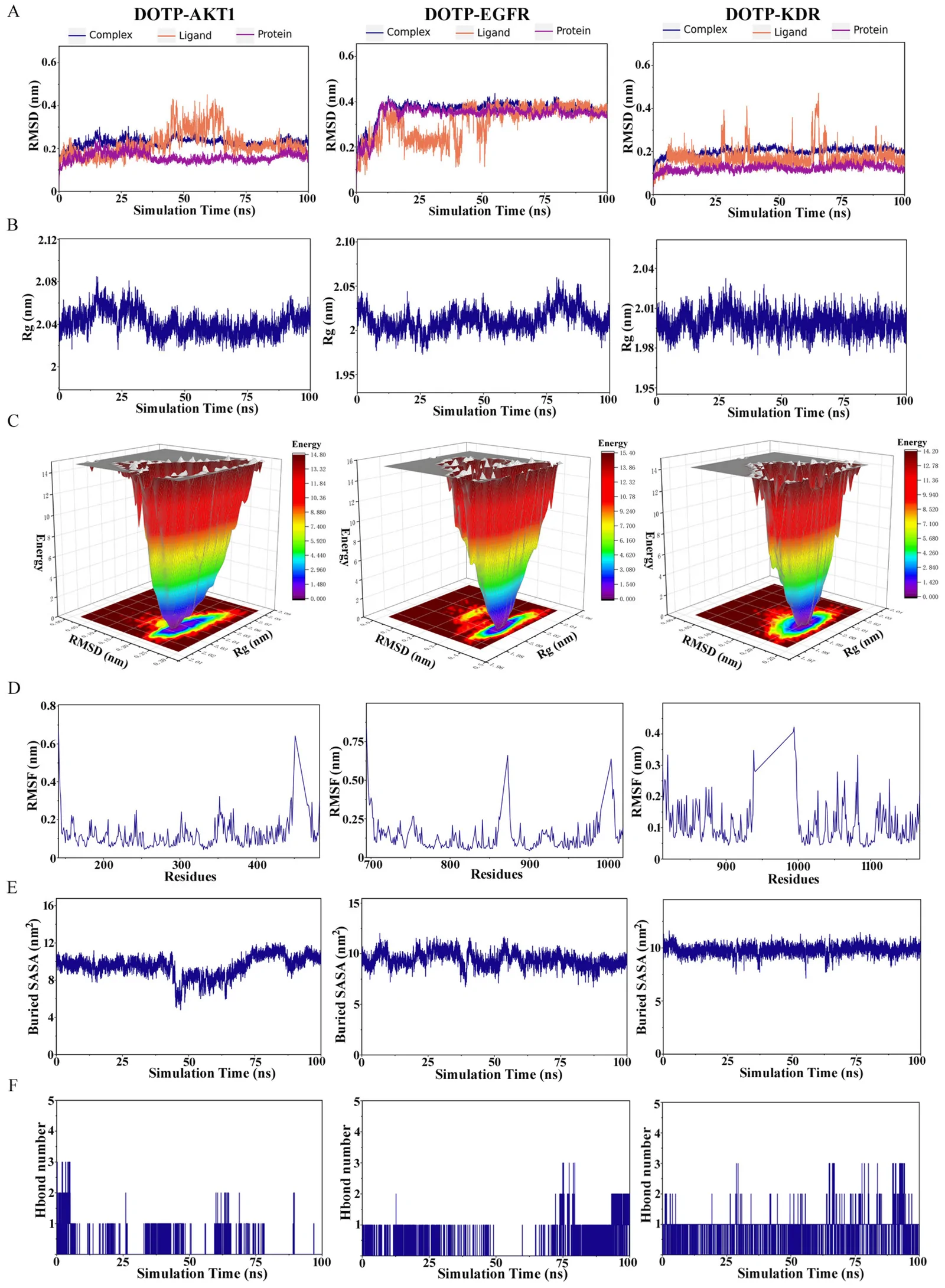
Molecular dynamics simulations of DOTP to the core targets. (**A**) RMSD value of the DOTP-target complexes over time. (**B**) Rg value of the DOTP-target complexes over time. (**C**) Three-dimensional free energy surface analysis of the DOTP-target complexes during simulation. (**D**) RMSF value of the DOTP-target complexes over time. (**E**) SASA dynamic changes in the DOTP-target complexes. (**F**) Hydrogen bond formation dynamics during molecular dynamics simulation. RMSD, root mean square deviation; Rg, radius of gyration; SASA, solvent-accessible surface area; RMSF, root mean square fluctuation.

To further verify the binding stability of DOTP with core targets, we employed molecular dynamics simulations to analyze key parameters including the root mean square deviation (RMSD), radius of gyration (Rg), free energy landscape (FEL), root mean square fluctuation (RMSF), buried solvent accessible surface area (buried SASA), hydrogen bond formation dynamics and residue contribution. The RMSD was utilized to assess the stability of the simulation system. The results showed that all the DOTP-protein complexes gradually became more stable as the simulation progressed (Figure 8A). Rg is an important parameter used to measure the overall structural compactness of protein-small molecule complexes. As illustrated in Figure 8B, the Rg values of the target proteins decreased and stabilized upon binding to DOTP, indicating that these interactions resulted in a more compact conformation. FEL was used to display the conformation of the complex and its relative stability. The results revealed that each of the three complexes exhibited one lower-energy state, indicating that the overall structures of these complexes were relatively stable (Figure 8C). Furthermore, the RMSF represents the degree of flexibility of the amino acid residues in proteins. The results showed that the flexibility of the amino acids around the DOTP was relatively low. Therefore, the DOTP-AKT1, DOTP-EGFR and DOTP-KDR complexes were more stable in terms of binding (Figure 8D). As a key measure of protein surface exposure, SASA was evaluated between the target proteins and DOTP in this simulation. The results indicated stable SASA values across the DOTP-AKT1, DOTP-EGFR, and DOTP-KDR complexes, demonstrating that DOTP did not substantially expand or contract after binding to these proteins (Figure 8E). In addition, the results of the analysis of hydrogen bond formation dynamics revealed that hydrogen bonds were formed between DOTP and the proteins AKT1, EGFR, and KDR (Figure 8F). In conclusion, all three core target proteins were stably bound to DOTP. Therefore, AKT1, EGFR and KDR target proteins may play crucial roles in DOTP-induced PAH, especially in terms of PAEC damage.

## 3. Discussion

In this study, network analysis, scTenifoldKnk virtual knockout technology, molecular docking, and molecular dynamics simulations were integrated to construct a DOTP-induced PAH framework. These findings demonstrated that AKT1, EGFR and KDR are target proteins for DOTP-induced PAH. The results revealed that DOTP-treated PAEC exhibited increased cellular damage and migration capacity, alongside elevated protein expression of AKT1, EGFR and KDR. Our data suggest that the common environmental disruptor DOTP may act as a critical risk factor contributing to the pathogenesis of PAH.

DOTP, as an environmentally friendly plasticizer, has completely replaced traditional toxic plasticizers. However, as research has progressed in recent years, it has become increasingly evident that long-term exposure to DOTP can also exert adverse effects by disrupting multiple cell signaling pathways [12,21,22,23]. More importantly, DOTP may be involved in the chronic inflammation pathway, thereby potentially increasing susceptibility to respiratory system diseases [13]. In this study, we revealed that DOTP may play a vital role in the pathological process of PAH through the regulation of reactive oxygen species pathway, HIF-1 signaling pathway, and MAPK signaling pathway. Notably, abnormal activation of reactive oxygen species, the HIF-1 signaling pathway, and the MAPK signaling pathway has been widely recognized as a key driver of vascular remodeling in PAH. By affecting these signaling pathways, DOTP may promote the development and progression of pulmonary vascular lesions.

We subsequently identified key genes for PAH associated with DOTP exposure, including AKT1, EGFR, KDR, and SRC. However, scTenifoldKnk analysis further indicated that AKT1, EGFR and KDR may be associated with network-level promotion of downstream PAEC damage and migration-related genes. We subsequently performed experimental validation. Assessment of LDH release, RT-qPCR, and Western blot analysis of ET-1 and eNOS revealed that DOTP has exerts toxic effects on PAECs, promoting inflammatory and endothelial-specific functional damage. Moreover, significant upregulation of AKT1, EGFR and KDR expression was observed in PAECs treated with DOTP, suggesting that AKT1, EGFR and KDR may be potential mediators of DOTP-induced PAH.

AKT1 is a crucial regulator of vascular remodeling and PAH and is involved in important cellular processes such as cell proliferation and migration [24]. Molecular docking results showed that DOTP has a good binding affinity of −5.5 kcal/mol for the AKT1 protein, which may activate the HIF-1 and MAPK signaling pathways, thereby promoting PAEC damage and migration. Molecular dynamics simulation results further indicated that the DOTP-AKT1 complex exhibited strong binding affinity and structural stability. This high-affinity binding is biologically meaningful, as it suggests that DOTP could act as an effective molecular modulator that targets AKT1 and perturbs its downstream signaling cascades, thereby participating in the pathological process of PAH.

The members of the EGFR family are vital signaling molecules that regulate cell proliferation and migration by activating the HIF-1, PI3K/AKT and MAPK signaling pathways in PAH [25,26]. Indeed, EGFR inhibitors have been proven to suppress vascular remodeling, highlighting the significant role of the EGFR family in PAH treatment [27]. Molecular docking and molecular dynamics simulation results showed that the binding of DOTP and EGFR was relatively stable. In addition, scTenifoldKnk analysis of PAECs identified that EGFR was the most influential hub within the endothelium, and GO enrichment directly targeted the core processes of PAH-related endothelial remodeling: endothelial cell migration and angiogenesis. Therefore, abnormal activation of EGFR under DOTP stimulation disrupts pulmonary vascular endothelial stability and ultimately promotes vascular remodeling and PAH progression.

KDR, also known as VEGFR2, is a receptor tyrosine kinase that serves as a key regulator of angiogenesis in PAH. Studies have shown that activation of the VEGFR2 signaling pathway leads to an increase in vascular permeability, ultimately resulting in distal vascular remodeling and PAH [28]. Inhibiting its activation can alleviate disease progression [29]. However, by contrast, complete inhibition of VEGFR2 exacerbates the occurrence and development of PAH, indicating that the VEGFR2 signaling pathway plays a multifaceted role in the pathogenesis of PAH [30,31]. This may be related to the complex pathological process of PAH. In this study, we showed that DOTP may bind to KDR and promotes its expression in PAECs. These findings suggest that the DOTP-mediated upregulation of KDR in PAECs may trigger downstream signaling cascades, thereby contributing to endothelial dysfunction and pathological angiogenesis in PAH.

Several limitations of the present study should be acknowledged. First, all downstream functional assays were performed at a single DOTP concentration of 200 μM. This concentration was selected based on the CCK8 results, at which DOTP induced cytotoxicity without causing massive cell death. However, this in vitro concentration may exceed the levels achieved under real-world human exposure, and a single-dose design cannot establish a clear dose–response relationship. Future studies incorporating multiple DOTP concentrations and, ideally, physiologically relevant exposure regimens are warranted to confirm the dose-dependent effects observed here. Second, our findings were derived exclusively from an in vitro PAEC model without animal model validation; therefore, the conclusions drawn here cannot be fully extrapolated to complex in vivo conditions. Third, although DOTP exposure was associated with upregulated AKT1, EGFR, and KDR expression alongside increased PAEC injury and migration, this study did not include gain- or loss-of-function interventions such as siRNA knockdown or specific inhibitor treatment. Consequently, the proposed regulatory mechanism requires further targeted validation. Fourth, according to previously reported affinity ranges for active small-molecule ligands, binding energies lower than −5.0 kcal/mol generally indicate favorable binding potential. Although the molecular-docking binding energies results (−5.5, −5.8, and −7.0 kcal/mol) suggest that the binding interactions between DOTP and the three proteins are relatively good, due to the lack of positive control comparisons, we cannot draw a clear conclusion on the relative binding affinity of DOTP compared to typical inhibitors. The molecular-docking binding energies are computational estimates rather than experimentally measured thermodynamic parameters, so reliable association constants therefore cannot be derived. Moreover, in vivo target engagement depends not only on intrinsic ligand protein binding affinity, but also on multiple additional factors, including the free-ligand concentration at the target site, tissue distribution, membrane permeability, metabolic stability, target protein abundance, and competition with endogenous ligands. Future studies incorporating multiple DOTP concentrations, animal-based in vivo experiments, targeted functional validation, and experimentally determined binding affinities are therefore warranted to consolidate the present findings.

## 4. Materials and Methods

### 4.1. Collection of DOPT Targets

DOPT was searched, identified, and verified in the PubChem database (https://pubchem.ncbi.nlm.nih.gov/ (accessed on 6 June 2026)) to obtain its structure, molecular formula, and SMILES format. Its targets were retrieved from the SwissTargetPrediction (http://www.swisstargetprediction.ch (accessed on 6 June 2026)), Superpred (https://prediction.charite.de/subpages/target_prediction.php (accessed on 6 June 2026)), and PharmMapper (https://www.lilab-ecust.cn/pharmmapper/ (accessed on 6 June 2026)) databases, with the species set to “Homo sapiens”. The results from the three databases were integrated, and deduplicated, and the target names were standardized using the UniProt database (https://www.uniprot.org/ (accessed on 6 June 2026)).

### 4.2. Identification of PAH-Related Targets

PAH-related targets were retrieved from the GeneCards (https://www.genecards.org/ (accessed on 6 June 2026)), OMIM (https://www.omim.org/ (accessed on 6 June 2026)), and GAD (https://maayanlab.cloud/Harmonizome/resource/Genetic+Association+Database (accessed on 6 June 2026)) databases, with the keywords set to “PAH”. The results from the three databases were integrated and deduplicated.

### 4.3. Intersection of DOPT Targets and PAH Targets

The intersection between DOPT targets and PAH disease targets was identified using a Venn diagram tool (https://www.bioinformatics.com.cn/ (accessed on 6 June 2026)).

### 4.4. Protein–Protein Interaction (PPI) Network and Core Target Identification

The intersecting target genes were submitted to the STRING database (https://string-db.org/ (accessed on 6 June 2026)) to construct the PPI network, with the species restricted to “Homo sapiens” and a minimum required interaction score of ≥0.4. The PPI network map was imported into Cytoscape_v3.7.1 (San Diego, CA, USA) for visualization. CentiScaPe (Verona, Italy) was used to calculate the topology parameters, and the cytoHubba plug-in was used to identify the core genes on the basis of MCC, MCODE, and CytoNCA.

### 4.5. Functional Enrichment Analysis

To investigate the potential biological functions and pathways shared by DOTP targets and PAH-related genes, we performed Gene Ontology (GO) annotation and Kyoto Encyclopedia of Genes and Genomes (KEGG) enrichment analyses by using the Hiplot Biomedical AI Platform (https://hiplot.com.cn/home/index.html (accessed on 7 June 2026)).

### 4.6. Molecular Docking and Dynamics Analysis

The 3D structures DOTP were retrieved from the PubChem database, and the target protein structures were obtained from the RCSB Protein Data Bank. The PDB numbers of the target protein are as follows: AKT1: 6CCY; EGFR: 8WD4; KDR: 3VO3. DOTP and protein structures were prepared using AutoDockTools_1.5.7 (La Jolla, CA, USA). Docking simulations were conducted to predict binding poses, calculate binding free energy (ΔG), and assess potential functional impact. ΔG < −5.0 kcal/mol suggests a stable interaction [32,33,34]. Molecular dynamics simulations were performed using GROMACS 2022.2. Proteins were modeled with the Amber14SB force field, and the solvent was TIP3P water. For small molecules, AM1-BCC charges were generated and GAFF2 atom types were assigned via Antechamber (https://ambermd.org/antechamber/ac.html (accessed on 15 June 2026)), followed by conversion to GROMACS topologies using ACPYPE (https://github.com/alanwilter/acpype (accessed on 15 June 2026)). The ion parameters were compatible with the TIP3P water model (Joung–Cheatham). Production simulations were conducted under NPT conditions for 100 ns with a time step of 2 fs and the Verlet cutoff scheme. After simulation, structural and interaction analyses were performed using the GROMACS tools, VMD (https://www.ks.uiuc.edu/Research/vmd/ (accessed on 15 June 2026)), and PyMOL 3.1.8 (https://pymol.org/ (accessed on 15 June 2026)). MM-PBSA binding free energy calculations were conducted using gmx_MMPBSA where necessary.

### 4.7. Single-Cell Sequencing Data Analysis and Virtual Knockout

A single-cell sequencing dataset (GSE210248), comprising samples from 3 normal donors and 7 patients with PAH, was downloaded from the GEO database, and included 22,704 cells and 19,582 genes. The Seurat package handled quality control and preprocessing of the gene expression matrix. The cell filtering criteria were an nFeature_RNA between 200 and 6000 and a percent.mt below 15%. The standardized method employed SCTransform v2 (Seurat v5). The clustering of cell clusters was carried out using the “FindNeighbors + FindClusters” method. Marker genes were identified using the FindAllMarkers function with the Wilcoxon rank-sum test. A total of 8021 marker genes were detected.

Afterward, using the scTenifoldKnk package (v1.0.3) as a network-based inference tool, we estimated gene network modifications in response to virtual perturbation. The screening criteria for differentially expressed genes were an FDR < 0.05 and a |log_2_(FC)| ≥ 1.5. Functional-annotation and gene-set enrichment analysis (GSEA) based on Z-score-ranked transcriptome data was performed using the clusterProfiler R package. Statistical significance thresholds were set at *p* < 0.05 and FDR < 0.05.

### 4.8. Cell Culture

Human PAECs were provided by Procell Life Science and Technology (Wuhan, China). The cells were cultured in DMEM enriched with 15% fetal bovine serum and 1% penicillin and streptomycin at 37 °C with 5% CO_2_ in humidified conditions. All experiments were performed using cells from the 4th to 6th passages. For the hypoxia exposure experiments, the cells were incubated in a Tri-Gas Incubator (Thermo, Waltham, MA, USA) in a water-saturated atmosphere containing 3% O_2_ and 5% CO_2_ for 24 h. For DOTP exposure, the cells were treated with DOTP at a concentration of 200 μM or with a DMSO vehicle control for 48 h.

### 4.9. Western Blot Analysis

Cellular protein was extracted using RIPA buffer (Beyotime Biotechnology, Shanghai, China) and separated on 10% or 12% SDS–PAGE gels. Afterward, the proteins were transferred to nitrocellulose filter membranes. The membranes were blocked with 5% nonfat milk at room temperature for 1 h. Anti–eNOS antibody (1:1000, 32027T, Cell Signaling Technology, Danvers, MA, USA), anti–ET1 antibody (1:500, bs-42253R, Bioss, Beijing, China), anti–AKT1 antibody (1:1000, 69471, GenuIN, Hefei, China), anti–EGFR antibody (1:1000, 69236, GenuIN, Hefei, China), and anti–KDR antibody (1:500, A00901-3, Boster, Wuhan, China) were used as the primary antibodies and incubated overnight at 4 °C. The membranes were subsequently incubated with HRP-conjugated secondary antibodies for 2 h at room temperature and visualized utilizing enhanced chemiluminescence reagents (Millipore, Burlington, MA, USA).

### 4.10. Cell Counting Kit-8 (CCK8) Assay

Approximately 5000 cells/well were cultured in 96-well plates and treated with DOTP at concentrations of 0, 25, 50, 100 and 200 μM or with a DMSO vehicle control for 48 h. Ten microliters of CCK8 reagent were added to each well, and the plates were incubated at 37 °C for 2 to 4 h. The absorbance at 450 nm was measured using a spectrophotometric microplate reader (Agilent BioTek, Winooski, VT, USA) to assess cytotoxicity.

### 4.11. Cell Immunofluorescence

The processed cell slides were subsequently fixed with 4% paraformaldehyde, permeabilized with 0.3% Triton X-100 (Beyotime Biotechnology, Shanghai, China), and blocked with 5% bovine serum. The cells were incubated with AKT1 (1:200), EGFR (1:100), and KDR (1:100) antibodies at 4 °C overnight and subsequently incubated with Cy3-conjugated goat anti-rabbit antibody for 2 h at 37 °C. DAPI was added to stain the nuclei at 37 °C. Finally, images were recorded using a confocal microscope system (Nikon, Tokyo, Japan). ImageJ—Pro Plus 6.0 was used to determine the mean fluorescence intensity.

### 4.12. Lactate Dehydrogenase (LDH) Release Assay

Approximately 5000 cells/well were cultured in 96-well plates according to the different experimental groups. LDH activity was measured according to the instructions of the LDH Release Assay Kit instructions (Beyotime Biotechnology, Shanghai, China).

### 4.13. Transwell Migration Assay

For the migration assay, cells were added to the top chamber of 24-well transwell chambers (8 μm; 3422; Corning, Corning, NY, USA). In brief, treated cells were resuspended in DMEM and placed in the upper compartment. The bottom chamber was filled with 5% FBS. After 24 h, the migrated cells were fixed with 4% paraformaldehyde and stained with 1% crystal violet. Random fields were imaged, and the number of cells was counted.

### 4.14. RNA Isolation and Quantitative Real-Time Polymerase Chain Reaction (RT-qPCR)

RNA was extracted by TRIzol (LABLEAD, Beijing, China) according to the manufacturer’s protocol. For each sample, 1 μg of total RNA was converted to cDNA using a Superscript First-Strand cDNA Synthesis Kit (HaiGene, Harbin, China), and the mRNA expression levels of TNF-α and IL-1β were detected via a real-time PCR system (TIANLONG (Xi’an, China)) with SYBR Green (TOYOBO, Osaka, Japan). Samples were normalized to β-actin expression via the 2^−ΔΔCT^ method. The sequences of primers used were as follows: TNF-α: forward (F), 5′-GACAAGCCTGTAGCCCATGT-3′, and reverse (R), 5′-GGAGGTTGACCTTGGTCTGG-3′; IL-1β: forward (F), 5′-CTCTTTGATGTCACGCACGATTTC-3′, and reverse (R), 5′-AGCCATCATTTCACTGGCGA-3′; β-actin: forward (F), 5′-AGAGCCTCGCCTTTGCCGATCC-3′, and reverse (R), 5′-CTAGAAGCATTTGCGGTGGACGAT-3′.

### 4.15. Data Analysis

The data were analyzed using GraphPad Prism 8.0 software (GraphPad Software Inc., La Jolla, CA, USA). The normality of the data was assessed using the Shapiro–Wilk test and the equality of group variance was assessed using Brown–Forsythe test before statistical testing. One-way ANOVA with Tukey’s post hoc test was used to compare multiple groups with equal variance, Brown–Forsythe and Welch ANOVA with Tamhane T2 post hoc tests were used to compare multiple groups with unequal variance, and the Kruskal–Wallis test followed by the Dunn posttest was used for multiple groups with nonnormally distributed data. *p* < 0.05 was considered to indicate statistical significance.

## 5. Conclusions

In summary, these findings not only extend our understanding of the potential toxicological consequences of long-term DOTP exposure, but also provide novel insights into the environmental risk factors contributing to PAH.

## Data Availability

The original contributions presented in this study are included in the article/Supplementary Material. Further inquiries can be directed to the corresponding authors.

## Supplementary Materials

The following supporting information can be downloaded at: https://www.mdpi.com/article/10.3390/ijms27188064/s1.
