# Supplementary material for: Exploring the Toxicological Impacts of DOTP Exposure on Pulmonary Arterial Hypertension via Network Toxicology, Virtual Knockout, Molecular Docking and Experimental Validation
Source: Int J Mol Sci. 2026 Sep 10;27(18):8064. doi: 10.3390/ijms27188064 (PMC13607242; doi:10.3390/ijms27188064)

Figure 6D

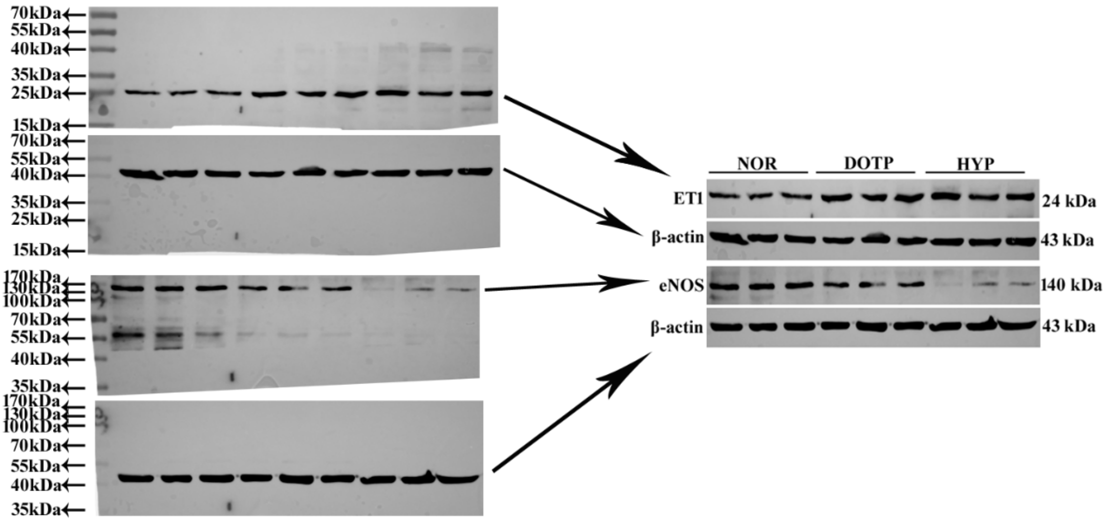

Figure 6F

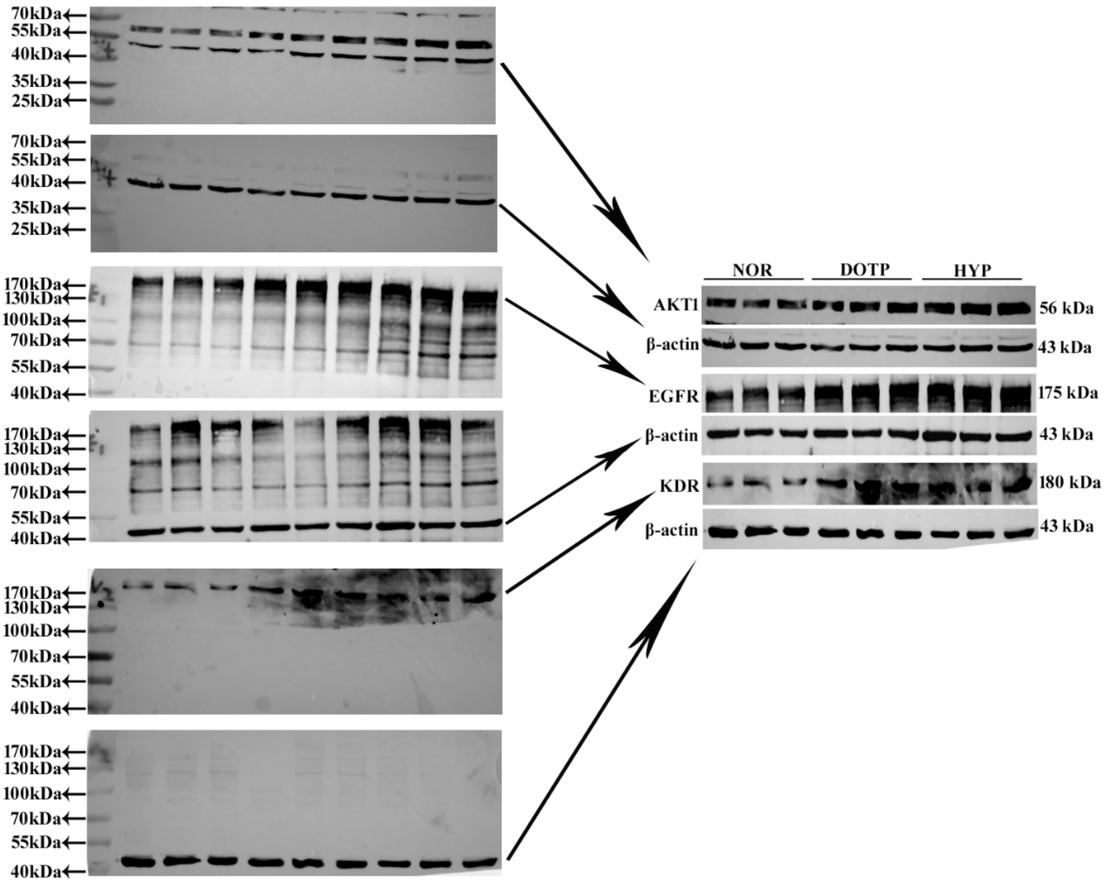

Supplement: Supplementary file 1 [file ijms-27-08064-s001.zip › ijms-4523296-supplementary.pdf]
